# Supplementary material for: Chromothripsis during telomere crisis is independent of NHEJ, and consistent with a replicative origin
Source: Genome Res. 2019 May;29(5):737–49. doi: 10.1101/gr.240705.118 (PMC6499312; doi:10.1101/gr.240705.118)
Supplement: Supplemental Material [file supp_gr.240705.118_Supplemental_file_1.zip › contigs/annotated_contigs/DB92/contig.2.DB92_length_216_mean_cov_5.55555555556.docx]

**DB92_length_216_mean_cov_5.55555555556**

```
TTTTGTTTCTATATGAATTA|ACATTCCCAGATAAAACCTGAGAAAATTTATT|AGACA|CTAGAGCTAGTGTGTCTGGTGACTGACAA
                     >chr14:104955911-104955943 + E=5e-02  >chr3:60922334-60922478 + E
TTTTCTAAAATTAACTGTTGTGTTAATAGCATTTAGATTTGTAAATTGAACTCCCCTTGCATAAACATTTAAACATTCAAAATAAAATC
=1e-66
TGTAAACTTTGGTAGCATGAACTCAA|GAGGATTCAGTCTTG
```
